# Novel Insights Into Refugia at the Southern Margin of the Distribution Range of the Endangered Species *Ulmus laevis*

**DOI:** 10.3389/fpls.2022.826158

**Published:** 2022-02-15

**Authors:** Sara Torre, Federico Sebastiani, Guia Burbui, Francesco Pecori, Alessia L. Pepori, Iacopo Passeri, Luisa Ghelardini, Alberto Selvaggi, Alberto Santini

**Affiliations:** ^1^Istituto per la Protezione Sostenibile delle Piante, IPSP-CNR, Florence, Italy; ^2^Dipartimento di Scienze e Tecnologie Agrarie, Alimentari Ambientali e Forestali (DAGRI), Università di Firenze, Florence, Italy; ^3^Istituto per le Piante da Legno e l’Ambiente - I.P.L.A. S.p.A., Turin, Italy

**Keywords:** conservation genetics, phylogeography, *Ulmus laevis*, plastome sequencing, forest trees, refugia, genetic polymorphisms

## Abstract

Riparian ecosystems, in long-time developed regions, are among the most heavily impacted by human activities; therefore, the distribution of tree riparian species, such as *Ulmus laevis*, is highly affected. This phenomenon is particularly relevant at the margins of the natural habitat of the species, where populations are small and rare. In these cases, it is difficult to distinguish between relics or introductions, but it is relevant for the restoration of natural habitats and conservation strategies. The aim of this study was to study the phylogeography of the southern distribution of the species. We sequenced the entire chloroplast (cp) genomes of 54 individuals from five sampled populations across different European regions to highlight polymorphisms and analyze their distribution. Thirty-two haplotypes were identified. All the sampled populations showed private haplotypes that can be considered an indicator of long-term residency, given the low mutation rate of organellar DNA. The network of all haplotypes showed a star-like topology, and Serbian haplotypes were present in all branches. The Balkan population showed the highest level of nucleotide and genetic diversity. Low genetic differentiation between populations was observed but we found a significant differentiation among Serbia vs. other provenances. Our estimates of divergent time of *U. laevis* samples highlight the early split of above all Serbian individuals from other populations, emphasizing the reservoir role of white elm genetic diversity of Serbian population.

## Introduction

Modern biodiversity conservation strategies rely, or at least should, on the knowledge of genetic resources (Gepts, 2006; Hoban et al., 2021). Big efforts were made in the exploration of genetic resources in the last decades (Halewood et al., 2018). In Europe, several foundation works were conducted on plant and animal organisms (Toro and Caballero, 2005; Koskela et al., 2013; Porth and El-Kassaby, 2014), exploring the geographical distribution and structure of genetic diversity and describing the patterns of reduction and expansion of populations mainly related to glaciation and recolonization, respectively (Petit et al., 2003; Heuertz et al., 2004; Mona et al., 2010; Lanier et al., 2015). In forest tree species, three main glacial refugia were identified in southern Europe, corresponding to the Iberian, Italian, and Balkan Peninsulas (Brewer et al., 2002; Heuertz et al., 2004; Magri et al., 2006), and post-glacial colonization routes of temperate species were hypothesized toward central and northern Europe (Petit et al., 2002; Magri, 2008). As a result of the expansion, a reduction in genetic diversity was expected from south to north (Gugerli et al., 2009), and this was partially confirmed, since most divergent populations were found in the Mediterranean areas, but the highest level of genetic diversity was observed in central Europe where the genetic lineages from different refugia got admixture (Petit et al., 2003). The picture that came out progressively gained complexity by considering the effect of preglacial variation distribution (Magri, 2008) and the existence of extra-Mediterranean refugia (Pedreschi et al., 2019). Despite these huge efforts, there are still species that are highly impacted by anthropogenic activity and by pests whose distribution is poorly investigated and that could represent a priority for habitat restoration.

European white elm (EWE) (*Ulmus laevis* Pall. = *Ulmus effusa* Willd. = *Ulmus pedunculata* Foug.) is a characteristic deciduous temperate forest tree species growing in river margins and lowland moist forests, and it can also tolerate dry soils of wooded steppe habitat in the central parts of the distribution. Seeds are generally anemochorous, but in the riparian habitats, hydrochory is an important means of transport, enabling long-distance dispersal (Vakkari et al., 2009). EWE is the only representative of the section *Blepharocarpus* in the Old World. *U. laevis* does not hybridize with other European species, namely, *Ulmus glabra* and *Ulmus minor* (Mittempergher and La Porta, 1991). Natural distribution range of EWE is mainly central and east European. It extends from Ural Mountains in the east to France in the west and from southern Finland in the north to Bosnia in the south (Svejgaard Jensen, 2003). There are even a few relict populations in south-western France (Timbal and Collin, 1999). Quite recently, some native relic scattered populations have been identified in the Iberian Peninsula (Fuentes-Utrilla et al., 2014). In Italy, it is generally considered allochthonous, naturalized in most regions (Pignatti et al., 2017) except in Piedmont (north–west) where it is considered native cryptogenic (Bartolucci et al., 2018) or native (Pignatti et al., 2017), in accordance with the hypothesis supported by regional botanic knowledge.

Elms in Europe are severely damaged by successive epidemic waves of Dutch elm disease (DED), a lethal disease caused by some non-native species of the genus *Ophiostoma*, namely, *Ophiostoma ulmi*, and *Ophiostoma novo-ulmi* ssp. *novo-ulmi* and *O. novo-ulmi* ssp. *americana* (Brasier, 2000). *U. laevis* is susceptible to the fungal agent of DED (*O. novo-ulmi*) (Santini et al., 2005), but it is able to avoid infections since it is far less attractive for the elm bark beetles, the main vector of the disease, than *U. minor* (Sacchetti et al., 1990). It is seldom attacked once other species are not present in an infested area anymore. Then, if the insect vectors do not change their feeding preferences, the genetic resources of *U. laevis* are not really endangered by DED (Collin, 2002). EWE populations are much more threatened by habitat destruction. Riparian forests, where EWE can be commonly found, are the sites of several human activities, namely, continuous cut for preventing floods, drained for reclaiming land for agriculture, and occasionally invaded by alien-human-introduced invasive plants. Therefore, the long-term survival of *U. laevis* throughout most of its distribution has been compromised (Franke et al., 2004). This has induced the generation of many small, isolated populations that result in vulnerability to genetic drift (Fuentes-Utrilla et al., 2014). In western Europe, the appropriate habitat for the species has been greatly reduced, although some small, isolated populations survive.

Typically, only populations that are recognized as native are included in the conservation process, while those that are considered introduced or have an uncertain status are not protected, which are excluded from regional Red Lists of the International Union for Conservation of Nature, even if they are rare and endangered (Decocq et al., 2004). The genetic tools used in most population genetic studies were based on different nuclear and molecular markers, mainly microsatellites (Morgante et al., 1996; Porth and El-Kassaby, 2014). The advent of high throughput sequencing offers from a few years the opportunity to explore genetic diversity at an unprecedented scale by sequencing portions to entire genomes and revealing unexplored genetic variation (Wang et al., 2001; Unamba et al., 2015; Ebrahimi et al., 2021).

Previous phylogeographic analyses in the genus *Ulmus* were based predominantly on neutral markers (Venturas et al., 2013; Zuo et al., 2020; Tamošaitis et al., 2021). In particular, the analysis of chloroplast (cp) markers in EWE populations (Whiteley, 2004) showed only three haplotypes, emphasizing the importance of exploring the entire genome. In recent years, the plastome analysis has facilitated molecular evolutionary studies because of its predominantly uniparental inheritance, small size, and slow nucleotide mutation rates (Wolfe et al., 1987), supplying the appropriate resolution frame to study plant phylogeography at deeper levels (Gitzendanner et al., 2018; Niu et al., 2018). Previous data on EWE genotyping highlighted the need to fill the gap on the southeast distribution of the species (Whiteley, 2004; Fuentes-Utrilla et al., 2014), even more so after the recent finding of an EWE stand in Italy, which was previously excluded from the natural distribution range. This work attempts to use whole plastid genome sequencing to better understand the phylogeography of *U. laevis* populations. The analysis of Serbian samples is critical to confirm the Balkans as the refugial origin of European populations.

Here, we aim to (a) explore the genetic structure of *U. laevis* at the south-western margin of its European distribution, (b) investigate whether Italian stands of white elms are natural or the result of anthropogenic introductions, and (c) discuss, in the light of new outcomes, the consequences for conservation and management strategies for this species.

## Materials and Methods

### Plant Material and DNA Extraction

The samples of *U. laevis* were collected in four areas and included in the natural range of the species: two in France (southwest and northeast), one in Spain, and one in Serbia.^1^ In addition, *U. laevis* samples were collected in northwestern Italy (Supplementary Table 1). Total genomic DNA was extracted from the leaves of each plant using the Invisorb Spin Plant Mini Kit (Invitek) following the instructions of the manufacturer.

### Plastome Sequencing and Assembly

Using a genome skimming approach, all the samples were subjected to DNA shotgun sequencing. Genomic libraries were prepared and sequenced in paired-end mode (2 × 150 bp reads) on an Illumina HiSeq 4000 platform (Novogene Co., Ltd., Beijing, China). Illumina reads were trimmed using Trimmomatic (Bolger et al., 2014) with the following options: trailing: 10, leading: 10, sliding window: 4:20, seed mismatches: 2; palindrome clip threshold: 30, simple clip threshold: 10, and minlen: 40. Clean reads of sample U1 (Supplementary Table 1) were used to assemble the cp genome of *U. laevis* using Novoplasty version 3.7 (Dierckxsens et al., 2017) with that of the congeneric species *Ulmus pumila* as reference (Zuo et al., 2017) and the following options: k-mer: 39 and seed: rbcL *U. laevis*. The software Snippy version 3.2-dev^2^ was used for reference-based mapping, consensus generation, and variant detection of all other samples. Gene annotation of plastomes was performed with GeSeq (Tillich et al., 2017) and BLAST (Altschul et al., 1990) analysis. The physical map of the *U. laevis* plastid genome was drawn with OGDRAW (Greiner et al., 2019).

### Characterization of cpDNA Polymorphisms, Haplotypic Network, and Genomic Diversity Analyses

Plastid sequences were aligned using MAFFT version 7 (Katoh and Standley, 2013), and the resulting alignment was revised manually using CLC Genomics Workbench version 8.5.1 (Qiagen Aarhus, Denmark). Haplotypes were defined as combinations of single nucleotide polymorphism (SNP) and indel (insertion-deletion) variants across the cp genomes. The estimates of haplotype and nucleotide diversity were performed with DnaSP version 6 (Rozas et al., 2017) and PEGAS (Paradis et al., 2015) in R 4.0.4 (R Core Team, 2021). GenoDive version 3.05 (Meirmans and Van Tienderen, 2004) was used to calculate genetic diversity indices. To estimate the relationships between haplotypes, a minimum spanning network of haplotype was constructed using PEGAS by considering all mutation events, both SNPs and indels. Population differentiation based on cpDNA haplotypes was evaluated using two parameters, *G*_ST_ and *N*_ST_ (PERMUT 2.0; Pons and Petit, 1996); the presence of phylogeographic structure is highlighted when significantly greater values of *N*_ST_ (that consider relatedness of haplotypes) in comparison with *G*_ST_ (that measure the only frequency of haplotypes) are obtained.

A hierarchical analysis of molecular variance (AMOVA; Excoffier et al., 1992) was evaluated using GenAlEx version 6.5 software (Peakall and Smouse, 2012): the molecular variance (Φ_PT_, an analog of *F*_ST_) subdivided into variation among populations and among individuals within populations was evaluated by a permutation test (*n* = 999). The software GenAlEx was used to perform principal coordinates analysis (PCoA), and then plotted in R with the package ggplot2 (Wickham, 2016).

The geographical structure of haplotype diversity was tested with a Bayesian approach implemented in BAPS 6.0 (Corander et al., 2008) using the “clustering with linked loci” analysis. We applied a spatial genetic mixture analysis to the predefined groups of individuals. The software uses Markov chain Monte Carlo (MCMC) simulation to group the sampled populations into K clusters predefined by the user, and the best partitioning is attained based on the highest marginal log-likelihood. After the testing stage, the final analysis was conducted for 10 replicates for K ranging from 2 to 10. Another clustering analysis was conducted by using the “Bayesian approach to phylogeographic clustering” (BPEC) package (Manolopoulou et al., 2016) implemented in R to verify the geographical structure of the populations and the most likely ancestral geographical locations. The analyses were conducted on a two-dimensional dataset (i.e., longitude and latitude), with three levels of parsimony relaxation (ds = 0, ds = 1, and ds = 2), allowing 5 migration events. Two MCMC chains were run for 100 million iterations.

### Phylogenetic Analysis of *Ulmus laevis* Populations and Estimation of Divergence Time

The cpDNA sequences of 54 *U. laevis* individuals and 13 plant species within the family Ulmaceae as outgroups (e.g., ten *Ulmus* species, two *Zelkova* species, and one *Hemiptelea* species) were aligned using MAFFT version 7.308 (Katoh and Standley, 2013). We created a time-calibrated phylogenetic tree, reconstructing the phylogeographical relationships and calculating the divergence time of *U. laevis* populations. The plastome phylogeny was inferred by applying the Bayesian inference (BI) method using the program BEAST2 version 2.6.3.0 (Bouckaert et al., 2014). The transversion model (TMV) substitution model was determined to be the best substitution model using a hierarchical likelihood ratio test framework as implemented in jModeltest version 2.1.10 (Darriba et al., 2012). After preliminary runs, we selected the best prior settings for various parameters to achieve an improved effective sample size (ESS) value. We used the lognormal relaxed molecular clock approach and assumed a Yule process of speciation, setting the parameters in BEAUti. The following five priors with normal distribution were selected for calibrations from published data (Zhang et al., 2021): (1) we constrained the crown age of the lineage Ulmaceae to be 85.39 million years ago (Mya) (sigma 1.0, offset: 0.0); (2) *Ulmus* clade was set to 52 Mya (sigma 1.0, offset: 0.0); (3) crown age of *Zelkova* was constrained to be 54.53 Mya (sigma 1.0, offset: 0.0); (4) crown age of *Hemiptelea* was set to 72.59 Mya (sigma 1.0, offset: 0.0); and (5) split between *Ulmus americana* and *U. laevis* was constrained to 17.5 Mya. The Bayesian posterior probabilities were sampled using the MCMC algorithm, which was run for 3.0 × 10^8^ generations and sampled every 1,000. The stationarity of the chains and convergence of MCMC simulations were monitored using Tracer version 1.7.1 (Rambaut et al., 2018), analyzing the log output of the BEAST2 analysis. ESSs of all parameters were more than 200, indicating that the estimations were confident. After stationarity was obtained, a maximum clade credibility (MCC) tree was generated using TreeAnnotator version 2.6.3.0 (Helfrich et al., 2019), setting the 20% of trees discarded as burn-in. The MCC tree was visualized using FigTree version 1.4.4 and then was plotted with a geological timescale using the strap package in R (Bell and Lloyd, 2015).

### Effective Population Size (N_*e*_)

We estimated the effective population size (*N*_*e*_) through time for the five populations, applying a non-parametric analysis based on the coalescence theory to cpDNA genome alignments for each population separately. The Bayesian skyline plot (BSP) approach was employed with the TMV substitution site model using a relaxed lognormal molecular clock with rate 1.0 and running 3.0 × 10^8^ MCMC simulations using the software BEAST2.

## Results

### *Ulmus laevis* Chloroplast Genome

**FIGURE 1:**
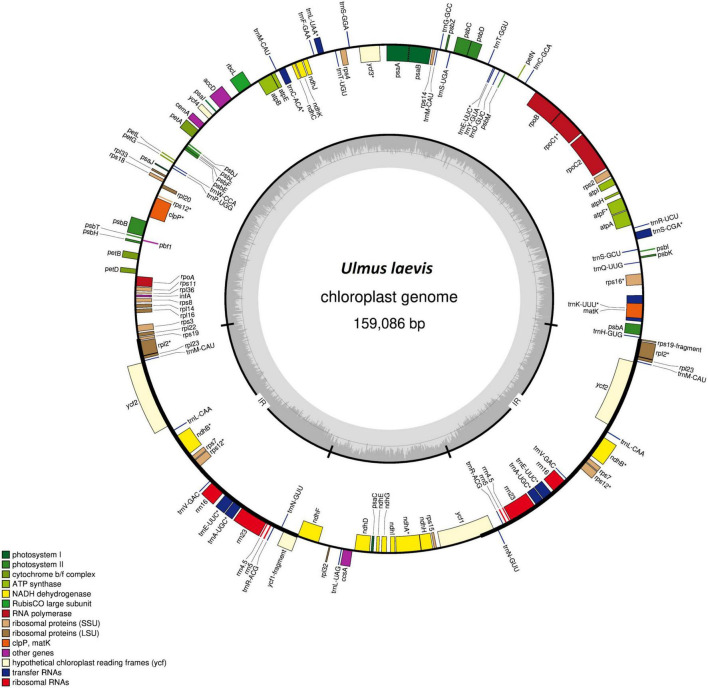
Gene map of the *Ulmus laevis* chloroplast (cp) genome. Annotated genes are colored according to the functional categories. Genes lying outside of the map were transcribed in the clockwise direction, while those inside the circle were transcribed counterclockwise. Small single copy (SSC), large single copy (LSC), and inverted repeats (IRs) are indicated in the inner circle. Genes with introns are marked with an asterisk.

The complete nucleotide sequence of the cp genome of *U. laevis* was determined through HTS “skim sequencing” (Kumar et al., 2021). Using 864,534 high-quality plastid reads, the cpDNA of one individual (Fsw1) was *de novo* assembled with a depth coverage of 815×. The cpDNA had a size of 159,086 bp, had a guanine-cytosine (GC) content of 35.6%, and its map had the typical quadripartite structure of angiosperms, including two inverted repeats (IRs) of 26,424 bp separated by a large single copy (LSC) of 87,594 bp and a small single copy (SSC) of 18,644 bp (Figure 1). The cp genome of *U. laevis* encodes 120 unique genes, of which 19 are duplicated in the IR, giving a total of 139 genes (Table 1). The Fsw1 cpDNA was used to map the sequencing reads (ranging from 88,686 to 322,795) of 55 other *U. laevis* samples with an average depth coverage of 304×.

**TABLE 1:** List of genes present in the *Ulmus laevis* chloroplast genome.

Category	Group of genes	Name of genes
Self-replication	Large subunit of ribosomal protein	*rpl2*, *14*, *16*, *20*, *22*, *23*, *32*, *33*, *36*
	Small subunit of ribosomal proteins	*rps2*, *3*, *4*, *7*, *8*, *11*, *12*, *14*, *15*, *16*, *18*, *19*
	DNA dependent RNA polymerase	*rpoA*, *B*, *C1*, *C2*
	rRNA genes	*rRNA 16S*, *23S*, *4.5S*, *5S*
	tRNA genes	*trnI-TAT*, *trnF-GAA*, *trnR-TCT*, *trnC-GCA*, *trnT-GGT*, *trnN-GTT*, *trnG-GCC*, *trnI-TAT*, *trnS-GGA*, *trnF-GAA*, *trnM-CAT*, *trnV-GAC*, *trnR-ACG*, *trnL-TAG*, *trnK-TT*, *trnN-GTT*, *trnL-CAA*, *trnI-CAT*, *trnV-GAC*, *trnR-ACG*, *trnL-CAA*, *trnN-GTT*, *trnL-CAA*, *trnI-CAT*, *trnP-TGG*, *trnW-CCA*, *trnT-TGT*, *trnfM-CAT*, *trnS-TGA*, *trnE-TTC*, *trnY-GTA*, *trnD-GTC*, *trnI-TAT*, *trnS-GCT*, *trnQ-TTG*, *trnK-CTT*, *trnH-GTG*
Photosynthesis	Photosystem I	*psaA*, *B*, *C*, *I*, *J*
	Photosystem II	*psbA*, *B*, *C*, *D*, *E*, *F*, *H*, *I*, *J*, *K*, *L*, *M*, *T*, *Z*
	Photosystem biogenesis	*pbf1*
	NadH oxidoreductase	*ndhA*, *B*, *C*, *D*, *E*, *F*, *G*, *H*, *I*, *J*, *K*
	Cytochrome b6/f complex	*petA*, *B*, *D*, *G*, *L*, *N*
	ATP synthase	*atpA*, *B*, *E*, *F*, *H*, *I*
	Rubisco	*rbcL*
Other genes	Maturase	*matK*
	Protease	*clpP*
	Envelop membrane protein	*cemA*
	Subunit Acetyl-CoA-Carboxylate	*accD*
	c-type cytochrome synthesis gene	*ccsA*
	Translation initiation factor IF-1	*infA*
Unknown	Conserved open reading frames	*ycf1*, *2*, *3*, *4*

The entire cp genomes of the 56 samples were aligned and haplotypes were identified. The dataset was then reduced to 54 cp genomes: samples U29 and U35 (Supplementary Table 1) were the same sample sequenced twice as well as sample U42 was sequenced twice as control for the sequencing process, as expected we obtained the same haplotypes from replicated samples. Only unique samples were kept for further analysis. The 54 plastomes were deposited in GenBank under the accession number PRJNA777116.

### Genetic Diversity and Population Differentiation

The alignment of the 54 cp genomes highlighted 190 nucleotide polymorphisms (14 SNPs and 176 indels). Most of the polymorphisms were neutral, and only 14 of these variations (6 SNPs and 8 indels) were in annotated regions. We found 3 mutations (SNPs) into coding sequences, 2 causing synonymous mutations (in *psaB* and *psaI* genes, respectively, both involved in Photosystem I), and 1 non-synonymous mutation in the *rpoC1* gene (that catalyzes a DNA-directed RNA polymerase).

All the polymorphisms allowed identifying 32 haplotypes (Supplementary Table 2). Twenty-four haplotypes were private, only 8 haplotypes were represented by more than one sample, and the most frequent haplotype included 11 samples (Table 2). The effective number of haplotypes varied across the four populations from 3.6 (France_SW) to 12.0 (Serbia). Haplotypic richness was close to the average for all four populations, ranging from 3.0 (France_SW) to 3.848 (Serbia). Private haplotypes were present in all five populations. The sequencing of the entire plastid genomes unveiled a high number of haplotypes that was reflected in high values of haplotypic diversity: total haplotype diversity was 0.945 and varied from 1.0 (Serbia, France_NE) to 0.801 (Italy). Interestingly, among other polymorphisms, we recovered a 7 bp insertion (TAATAAA) already described in the study by Fuentes-Utrilla et al. (2014) in 5 out of 10 Spain samples and 1 sample from France_SW. Overall nucleotide diversity (π = 1.028 × 10^–5^) was low and ranged from the higher value 2.439 × 10^–5^ in Serbia to 0 in Spain, where all variants were indels. A comparison of diversity indices obtained with SNPs and indels is reported in Supplementary Table 3.

**TABLE 2:** Sample size in each population (N), number of haplotypes detected in each population (A), number of private haplotypes (P), effective number of haplotypes (N_e), and genetic diversity suggested by Nei (1987) corrected for sample size (hapDiv) and nucleotide diversity (nucDiv) in chloroplast DNA sequences of *Ulmus laevis* populations.

Population	N	A	P	N_e	hapDiv	nucDiv
France_SW	5	4	2	3.6	0.900	6.286e-06
France_NE	8	8	6	8.0	1.000	3.143e-06
Serbia	12	12	10	12.0	1.000	2.439e-05
Italy	19	9	5	4.1	0.801	1.764e-06
Spain	10	6	5	5.5	0.911	NA

**FIGURE 2:**
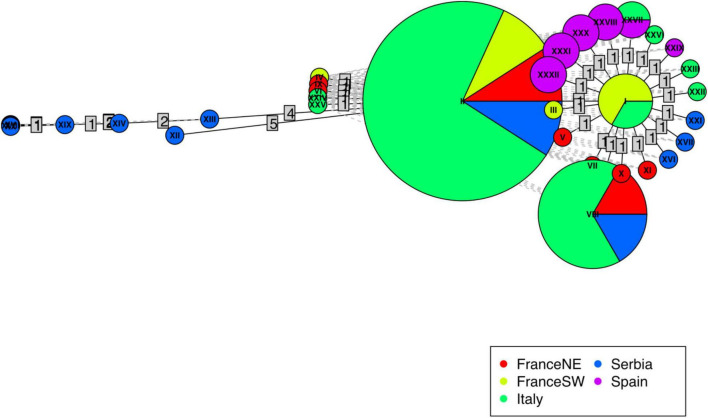
Haplotype network of the 32 *Ulmus laevis* haplotypes computed using R package “pegas.” Circles are colored according to the provenance of haplotype from different geographic areas. The size of the circles is proportional to the frequencies of each haplotype across all populations. Lines represent the mutational steps between haplotype sequences.

The median-joining network of all haplotypes (Figure 2) showed a star-like topology consisting of a central haplotype (I) from which most of the other haplotypes radiated and separated usually by one to two mutations. Serbian haplotypes were present all over the network, close to the center but also with the most diverging haplotypes (XIX, XX).

Total genetic diversity was high (*H*_*T*_ = 0.960), but the largest part of cpDNA variation was found within the populations (*H*_*S*_ = 0.922). The analyzed *U*. *laevis* populations showed the low levels of population differentiation (*G*_*ST*_ = 0.050). Low but significant differentiation was observed among the pairs Italy–Serbia, Spain–Serbia, and Spain–Italy (Table 3). In addition, the AMOVA showed that the differences among populations explained 31% of the total variation, while most of the variance (71%) was located within populations (data not shown).

**TABLE 3:** Values of *G*_ST_ suggested by Nei (1987) for all pairs of populations below the diagonal and *N*_ST_ above.

	France_SW	France_NE	Serbia	Italy	Spain
France_SW	–	0.008	0.308	−0.061	0.012
France_NE	0.023	–	0.353*	−0.033	0.346*
Serbia	0.030	−0.021	–	0.351*	0.339*
Italy	0.054	0.027	0.049	–	0.311
Spain	0.094	0.045	0.044	0.139	–
All samples G_ST_	0.034				
All samples N_ST_	0.078				

Tests of phylogeographical signal (i.e., N_ST_ > G_ST_): *p < 0.05.

**FIGURE 3:**
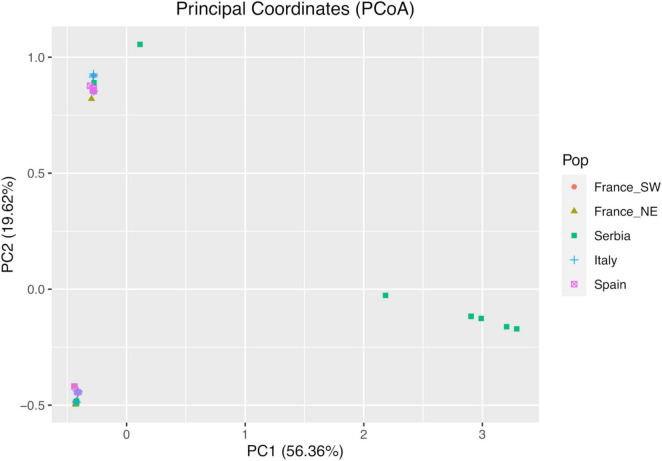
Principal coordinates analysis (PCoA) showing variation between the 54 chloroplast (cp) genomes. Each point represents a haplotype. Geographical regions are marked by different colors.

The PCoA scatterplot of the 54 cp genomes is presented in Figure 3. The first two PCoA axes account for about 76% of the total variation in the cpDNA, revealing a clear clustering in three groups with multiple haplotypes. The group at the extreme right of the first axis included only Serbian haplotypes. In addition to the three groups, another sample from Serbia (Se14) was resolved.

**FIGURE 4:**
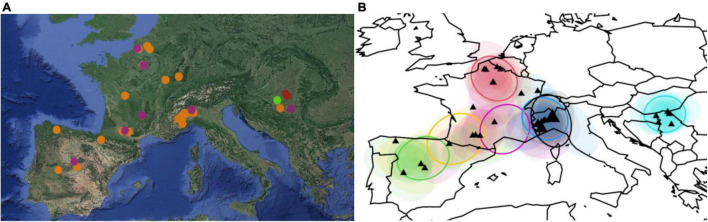
**(A)** Map showing the location of the four genetic clusters identified by Bayesian analysis of population structure (BAPS) for *k* = 4, using the spatial model. Chart was generated by PhyloGeoViz (Tsai, 2011). Terrain image copyright by Google Earth. The colors represent the cluster to which the analyzed individuals belong based on BAPS assignment. **(B)** Cluster analysis built by the Bayesian phylogeographic and ecological clustering. Each colored areas indicate a different phylogeographic grouping.

Using the Bayesian methods, optimum genetic clusters (*k* = 4) were obtained, which did not correspond to the populations. The analysis with BAPS did not display a strong genetic structure along the western part of the sampled species distribution, and two major clusters contained individuals from all four populations (i.e., Spain, France_SW, France_NE, and Italy); nevertheless, two clusters were represented only in Serbia: one with few individuals and the other one included only one individual (Figure 4A).

The BPEC clustering showed high uncertainty about the location and number of clusters in the assignment of plastid haplotypes, except for Serbian individuals (Figure 4B): haplotypes were assigned to “Serbian” phylogeographic cluster with generally high posterior probabilities (0.64–0.98). The most likely ancestral locations corresponded to sites located in the Ticino valley.

### Molecular Dating

**FIGURE 5:**
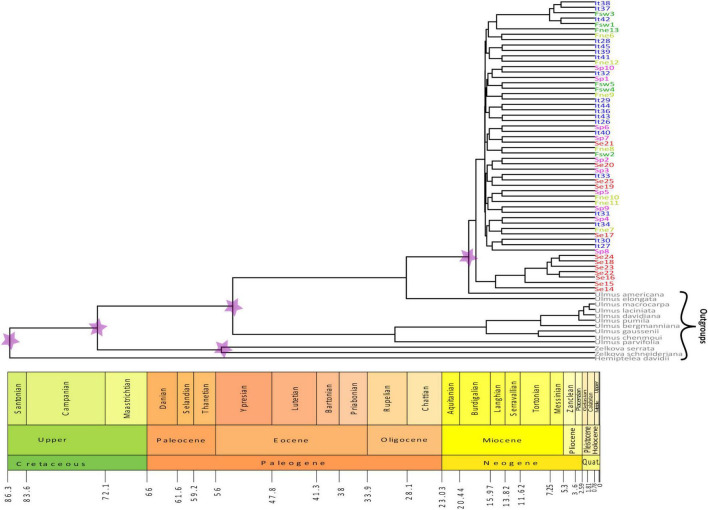
Phylogenetic tree of *Ulmus laevis* individuals calculated using BEAST software. The tree includes 54 *U. laevis* samples from five populations (indicated by different colors: blue = Italy, dark green = France_SW, light green = France_NE, red = Serbia, pink = Spain) and 13 outgroups species within Ulmaceae family: *Ulmus americana* (NC_044473.1); *Ulmus elongata* (NC_046061.1); *Ulmus macrocarpa* (NC_032720.1); *Ulmus laciniata* (MT742253.1); *Ulmus davidiana* (NC_032718.1); *Ulmus pumila* (MW279236.1); *Ulmus bergmanniana* (NC_057601.1); *Ulmus gaussenii* (NC_037840.1); *Ulmus chenmoui* (NC_037758.1); *Ulmus parvifolia* (MT701612.1); *Zelkova serrata* (NC_040958.1); *Zelkova schneideriana* (NC_041074.1); and *Hydrangea davidii* (MK070168.1). The stars on nodes indicate the five points of age constraints on divergent time. Time axis shows the age in Mya. Geological time scale visualized with the R strap package.

The Bayesian phylogenetic inference of cpDNA haplotypes subdivided populations into two main clades, one associated with almost all samples from Serbian populations (Se14, Se15, Se23, Se16, Se22, Se18, and Se24; clade I) and another one composed of other samples (clade II) (Figure 5). The molecular dating analysis suggested that the two clades diverged during the Upper Miocene, 17.3 Mya ago [95% highest posterior density (HPD): 12.9–18.7]. Lineages from the Serbian region constituting clade I show more recent allelic divergence, in the range of 5.1–10.1 Mya (95% HPD: 0–6.2 and 3.8–16.7, respectively). In clade I, the Se14 sample started diverging before the others, exhibiting greater genetic differentiation, as confirmed by the other analyses. Regarding clade II, it is noteworthy a subclade including five samples (i.e., It42, Fsw3, It38, Fsw1, and It37) from Italian and French_SW populations that also show a relatively recent divergence time, from 4.9 to 5.96 Mya (95% HPD: 0.01–6.1 and 0.1–8.0, respectively). Among all 13 outgroups, *Hemiptelea* was confirmed to be the most genetically divergent, and this is consistent with earlier studies (Ueda et al., 1997; Sytsma et al., 2002; Zhang et al., 2021), being a sister of the *Ulmus* and *Zelkova* genera.

**FIGURE 6:**
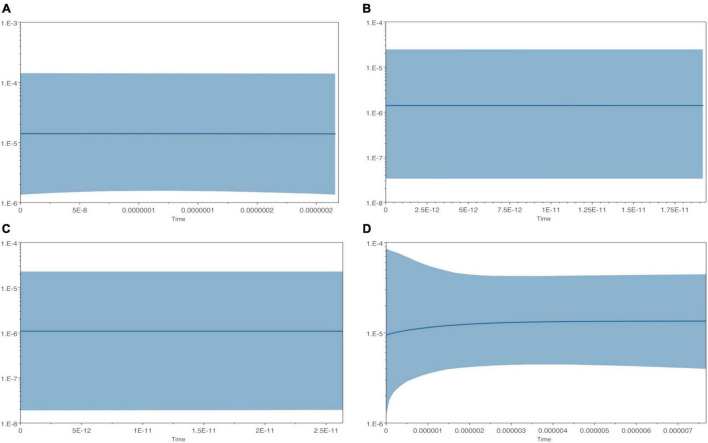
Bayesian Skyline plots derived from cpDNA sequences of *Ulmus laevis*. The analysis was performed for each population separately. The *X*-axis is in units of million years ago (Mya) and the *Y*-axis is the population size, expressed in units of Neτ, the product of effective population sizes per generation length. The thick solid line represents the mean effective population size *N*_*e*_, while the 95% highest posterior density (HPD) limits are shown by the blue area. **(A)** France_NE, **(B)** France_SW, **(C)** Italy, and **(D)** Serbia.

Profiles for the effective population size (*N*_*e*_) are shown in the four Skyline plots in Figure 6 (it was not possible to calculate a Skyline plot for the Spanish population due to the lack of informative variations). The BSPs suggest that the median estimate of population size of all four European populations remained constant throughout the time. Only the Serbian population revealed a quite small increase in the effective population size (*N*_*e*_) just after the coalescence, while no significant changes in *N*_*e*_ were observed in the other populations. French (NE and SW) and Italian populations share a stable ancestral size.

## Discussion

This study sought to explore the genetic structure of the species *U. laevis* in the southern part of its distribution range. Two main focuses were identified, namely, (i) the unexplored glacial refugium in the Balkan Peninsula and (ii) whether small Italian stands in the north-west (Piedmont and Ticino valley) are relic native ones or allochthonous.

This study is among the first to use large-scale intraspecific plastome sequencing of a European forest tree species to explore genetic diversity at population level. The plastid genome of *U. laevis* was recently sequenced (Zhang et al., 2021), but this study provides the first complete annotated plastid genome of EWE and the assessment of intraspecific diversity together with the elucidation of the phylogeography of this species in its southern European range. The cp markers have long been used in forest tree population genetics but were often limited to few polymorphic loci (Scotti et al., 2008; Bagnoli et al., 2009; Dias et al., 2020). The cpDNA sequences of *U. laevis* were highly conserved in terms of size, gene number, and arrangement; however, the full cpDNA sequencing of *U. laevis* unveiled unexplored genetic variation. In fact, 190 polymorphic sites were identified that gave rise to 32 haplotypes, and all the sampled populations showed private haplotypes.

Paleobotanical records witnessed the presence of *Ulmus* spp. in Italy (Mercuri et al., 2002; Amorosi et al., 2014) but, unfortunately, these reports cannot shed light on the past white elm distribution in Italy because, so far, it is not possible to distinguish *Ulmus* species. Our estimates of divergent time of *U. laevis* samples highlight that a subset of Serbian individuals started diverging from the Upper Miocene, while another subset formed a clade with all other provenances. Considering that this approach produces values regarding allelic divergence, while the species are always more recent, our tree is consistent with the divergent times calculated for the other Ulmaceae species (Zhang et al., 2017; Hou et al., 2020). The early split of above all Serbian individuals from other populations combined with the diversity that cluster I samples exhibit in more recent times highlights the role of the Serbian population as a reservoir for EWE genetic diversity and main source for recolonization. At the same time, the more recent divergence exhibited by samples from Italy and southwest France could suggest their long-term refugia isolation.

Considering that the mutation rate of organellar DNA is remarkably low (the mutation rate of cpDNA is in the order of 1.3 × 10^–9^) (Wolfe et al., 1987), private haplotypes (P) can be considered an indicator of long-term residency which makes anthropogenic introductions unlikely (Wares et al., 2002). Interestingly, the higher number of P, as well as the highest effective number of haplotypes, was observed in Serbia. Nevertheless, private haplotypes were found also in all other areas, though at reduced frequency.

The high number of private haplotypes is reflected in the haplotype network that, alongside the star-like structure, has many tips. The central haplotype, haplotype I, common to France_SW and Italy, should represent the most ancient condition, according to the coalescent theory (Castelloe and Templeton, 1994), while more recently derived haplotypes should be at the tips. According to Posada and Crandall (2001), the haplotypic structure would predict Italy and France_SW as potential refugia but most of the haplotypes are close to haplotype I, differing for just one mutation; hence, this structure is not so strong. In fact, the haplotypes found in the Serbian population are distributed over the entire network, from close derivatives of ancestral haplotypes to most distant, and their frequent appearance is remarkable and in agreement with a long-term occurrence.

Genetic diversity among and within populations is expected to be higher in refugia than in recently colonized areas. Plant populations in refugia are recognized as those that exhibit high levels of genetic diversity and uniqueness (Petit et al., 2003; Carnaval et al., 2009). Haplotype diversity is high in all samples, but interestingly, Serbian population has higher nucleotide diversity (π = 2.439e-05). Thus, higher genetic diversity evidenced by the Serbian population could reflect the accumulation of nucleotide mutations that characterize the isolation of individuals belonging to a refugia population. The Balkan Peninsula might have provided suitable habitat for *U. laevis* during the Pleistocene climate oscillations. Multiple refugia have been inferred in the Balkan Peninsula for many animals and plants during the Pleistocene glacial periods (Ursenbacher et al., 2008; Bagnoli et al., 2016).

Our phylogeographic survey showed a low genetic differentiation due to the high level of haplotype diversity found within populations. Overall, *N*_*ST*_ was not significantly higher than *G*_*ST*_; however, no negligible phylogeographic signal was detected in pairwise comparisons. In fact, *N*_*ST*_ of Serbian samples was significantly higher than *G*_*ST*_ of samples from Italy, Spain, and France_NE.

The Bayesian genetic clustering identified four groups, all of which present in Serbia, while only two were detected in the other sampled areas; these data together with the results of PCoA highlight the Balkan area as the one of the greatest diversity, which is possibly the main refuge area for the species. The confirmed absence of a clear structure is particularly significant considering the use of uniparentally inherited genetic markers. In contrast, the weak genetic structure within regions can be explained by a high rate of seed dispersal and long-range colonization of plastome variants (maternally inherited with seeds), while the overall observed genetic structuring could be originated by the accumulation of mutations in different refugia followed by recent population expansion. Consistent with these observations are the results of BSPs used for inference of coalescence patterns (Heller et al., 2013), which indicate a peculiarity of Serbian individuals attributable to the glacial refuge condition.

According to Fuentes-Utrilla et al. (2014), Spain can be considered a potential refuge, but in this view, Italy emerged as a previously unrecognized native population. If the appearance of new alleles following introductions can be ruled out because of the very low mutation rate and then two alternative hypotheses can be formulated, namely, the first one is that the observed data are the product of multiple introductions, but the sampling was not adequate to highlight the source; alternatively, we are observing the results of a composite demographic history, with small/cryptic refugia scattered at the margins of actual species distribution contributing to the actual diversity of haplotypes. Although we are dealing with a small-scale sample size, it was established that this should not interfere with the estimates of patterns of genetic diversity at a wide geographical range (Taberlet et al., 2012). Overall, the observed pattern of diversity could be explained with the existence of a principal refuge in the Balkans that represented the main source of post-glacial colonization from southeast to central and northern Europe. Based on our results, mainly the presence of private haplotypes, the Italian population may no longer be considered an anthropogenic introduction, but could be considered native and, consistently, with previous study for French (Timbal and Collin, 1999) and Spanish populations (Fuentes-Utrilla et al., 2014), all most likely originated from Serbian refuge.

Although the connection with Balkan refugia still needs deeper investigation, unfortunately, there are no warnings about the presence of white elms on the northeastern part of Italy, but long-range dispersal cannot be ruled out.

In support of our hypothesis, there are also arguments linked to the ecology of the species and the habitat transformation: most of the sampled trees were at the margin of high intensively cultivated areas close to rivers or scattered not far from such small stands. The area where we hypothesized a native presence of white elms, on the western side of the Po valley, experienced a high rate of population growth since the Neolithic Age. During the Roman Age, an extensive deforestation occurred, due to the assignment of land grants to war veterans, and continued in different ways till nowadays (Marchetti, 2002). The prolonged human presence highly impacted natural ecosystems by mainly extending urbanization and cultivated areas of agricultural crop. In that context, the introduction of white elms, species with no great economical relevance, especially at the bottom of the Alps, has no reasonable explanations. The Italian EWE population, which is conserving an important amount of genetic diversity, is composed of small, scattered populations at the margin of the distribution of species, and this pattern is in line with the evolution of the territory. Continued anthropogenic action along river courses could further limit the current populations, putting them at risk of extinction. This risk is a call for the maintenance of evolutionary processes through the conservation of genetic resources. Riparian forests, in whose ecosystem the EWE is a distinctive species, have high biodiversity and an important ecological role, so any change in their ecological dynamics also impacts the population dynamics of trees and, thus, their genetic diversity (Guilloy-Froget et al., 2002). For this reason, the conservation of genetic resources should be done with a dynamic approach not only at the level of single species but also on the whole riparian ecosystem (Lefèvre et al., 2013).

The pattern of genetic diversity observed in *U. laevis* is largely congruent with other previously studied European species, such as *Hordelymus europaeus* and *Fagus sylvatica* and another riparian tree, *Alnus glutinosa* (Magri, 2008; Dvořková et al., 2010; Havrdová et al., 2015); in particular, it points to the existence of a main refugium in the Balkan Peninsula, while additional southern refugial rear-edge populations in Iberian and Italian Peninsulas represent current the relicts with unique haplotype diversity.

Although more intensive sampling of the European populations in combination with nuclear genotyping is needed to give a more complete picture of the phylogeography of *U. laevis*, this study, to the best of our knowledge, for the first time extended the population genetic analysis of EWE to Italian and Balkan populations, revealing probably the main refuge of the species during the last glaciation. In addition, we extended the natural range of *U. laevis* in Europe to northwestern Italy and highlighted their small size and fragmentation and also at risk. The risk comes more from population size and habitat erosion than from DED; anyway, proper management practices become important to preserve these small stands with both *in situ* and *ex situ* actions to preserve and hopefully restore riparian ecosystems.

## Data Availability Statement

The datasets presented in this study can be found in online repositories. The name of the repository and accession number can be found below: https://www.ncbi.nlm.nih.gov/genbank/, PRJNA777116.

## Author Contributions

FS and ASa conceived the study. ST, FS, IP, and AP conducted all laboratory and population genetics analyses. GB, FP, ASa, LG, and ASe collected the samples. ST and FS led the writing of the manuscript. All authors contributed to the article and approved the submitted version.

## Conflict of Interest

The authors declare that the research was conducted in the absence of any commercial or financial relationships that could be construed as a potential conflict of interest.

## Publisher’s Note

All claims expressed in this article are solely those of the authors and do not necessarily represent those of their affiliated organizations, or those of the publisher, the editors and the reviewers. Any product that may be evaluated in this article, or claim that may be made by its manufacturer, is not guaranteed or endorsed by the publisher.

## Footnotes

1
http://www.euforgen.org/species/ulmus-laevis/

2
https://github.com/tseemann/snippy
