# Supplementary material for: Novel Insights Into Refugia at the Southern Margin of the Distribution Range of the Endangered Species Ulmus laevis
Source: Front Plant Sci. 2022 Feb 15;13:826158. doi: 10.3389/fpls.2022.826158 (PMC8886209; doi:10.3389/fpls.2022.826158)
Supplement: Supplementary file 1 [file Table_1.docx]

**Supplementary Table S1** Identification code and location information for analysed samples. Latitude and longitude are given in degrees, minutes and seconds.

| **Population code** | **Sequence code** | **Sample code** | **Sample location** | **Latitudine** | **Longitudine** |
| --- | --- | --- | --- | --- | --- |
| **France_SW** | U1 | Fsw1 | Fiac | 43°42'8" | 1°43'6" |
|  | U3 | Fsw2 | Grenade-sur-Garonne | 43°46'28" | 1°17'38" |
|  | U4 | Fsw3 | La Trimouille | 46°28'1" | 1°2'26" |
|  | U5 | Fsw4 | Saint-Georges | 45°1'18" | 3°7'51" |
|  | U6 | Fsw5 | Merville | 43°43'23" | 0°59'46" |
| **France_NE** | U2 | Fne6 | Locquignol | 50°11'58" | 3°43'6" |
|  | U7 | Fne7 | Colmar | 48°4'44" | 7°21'20" |
|  | U8 | Fne8 | Wavrans-sur-l'Aa | 50°11'58" | 2°8'11" |
|  | U9 | Fne9 | Hem-Hardinval | 50°9'47" | 2°18'14" |
|  | U10 | Fne10 | Vitrey-Sur-Mance | 47°48'55" | 5°45'45" |
|  | U11 | Fne11 | Hasnon | 50°25'19" | 3°23'9" |
|  | U12 | Fne12 | Pierre-Levee | 48°54'2" | 3°2'19" |
|  | U13 | Fne13 | Villemareuil | 48°55'19" | 2°58'25" |
| **Serbia** | U14 | Se14 | Apatin | 45°38'30" | 18°56'58" |
|  | U15 | Se15 | Padej | 45°50'41" | 20°13'13" |
|  | U16 | Se16 | Kanjiza | 46°02'31" | 20°04'45" |
|  | U17 | Se17 | Belgrado | 44°49'12" | 20°26'12" |
|  | U18 | Se18 | Padej | 45°50'41" | 20°13'13" |
|  | U19 | Se19 | Morovic | 44°59'37" | 19°10'03" |
|  | U20 | Se20 | Apatin | 45°38'30" | 18°56'58" |
|  | U21 | Se21 | Padej | 45°50'41" | 20°13'13" |
|  | U22 | Se22 | Kanjiza | 46°02'31" | 20°04'45" |
|  | U23 | Se23 | Kanjiza | 46°02'31" | 20°04'45" |
|  | U24 | Se24 | Kanjiza | 46°02'31" | 20°04'45" |
|  | U25 | Se25 | Kanjiza | 46°02'31" | 20°04'45" |
| **Italy_Lombardia** | U26 | It26 | Bernate Ticino A | 45°28'59" | 8°48'9" |
|  | U27 | It27 | Bernate Ticino A | 45°28'59" | 8°48'8" |
|  | U28 | It28 | Bernate Ticino A | 45°28'59" | 8°48'7" |
|  | U29 | It29 | Cuggiono | 45°29'45" | 8°48'3" |
|  | U30 | It30 | Bernate Ticino A | 45°28'58" | 8°48'4" |
|  | U31 | It31 | Vigevano, Sforzesca | 45°17'0" | 8°53'56" |
|  | U32 | It32 | Bereguardo | 45°15'6" | 9°1'10" |
|  | U33 | It33 | Bernate Ticino B | 45°29'4" | 8°48'4" |
|  | U34 | It34 | Bernate Ticino B | 45°29'4" | 8°48'6" |
|  | U35 |  | Cuggiono | 45°29'45" | 8°48'3" |
| **Italy_Piemonte** | U36 | It35 | Luserna San Giovanni | 44°49'7" | 7°16'4" |
|  | U37 | It36 | Crescentino | 45°11'21" | 8°05'51" |
|  | U38 | It37 | Isolotto del Ritano | 45°14'3" | 7°59'49" |
|  | U39 | It38 | IPLA, Torino | 45°05'15" | 7°44'24" |
|  | U40 | It39 | Isolotto del Ritano | 45°14'3" | 7°59'49" |
|  | U41 | It40 | Racconigi | 44°46'27" | 7°41'18" |
|  | U42 | It41 | Racconigi | 44°46'31" | 7°41'23" |
|  | U43 | It42 | Polonghera | 44°49'38" | 7°37'1" |
|  | U44 | It43 | Baudenasca | 44°51'2" | 7°23'00" |
|  | U45 | It44 | Bacino del Pesio, Oasi di Crava Morozzo | 44°25'13" | 7°43'28" |
| **Spain** | Us1 | Sp45 | Jaraiz de la vera | 40°03'37'' | 5°45'18'' |
|  | Us2 | Sp46 | Jaraiz de la vera | 40°03'37'' | 5°45'18'' |
|  | Us3 | Sp47 | Palazuelos de Eresma | 40°55'59'' | 4°03'00'' |
|  | Us4 | Sp48 | Palazuelos de Eresma | 40°55'59'' | 4°03'00'' |
|  | Us5 | Sp49 | Oroz-Betelu | 42°53'47'' | 1°18'11'' |
|  | Us6 | Sp50 | Oroz-Betelu | 42°53'47'' | 1°18'11'' |
|  | Us7 | Sp51 | Cangas de Narcea | 43°10'17 | 6°32'20'' |
|  | Us8 | Sp52 | Cangas de Narcea | 43°10'18 | 6°32'20'' |
|  | Us9 | Sp53 | Valdelatas | 40°32'04'' | 3°40'47'' |
|  | Us10 | Sp54 | Valdelatas | 40°32'04'' | 3°40'47'' |
