# Supplementary material for: Novel Insights Into Refugia at the Southern Margin of the Distribution Range of the Endangered Species Ulmus laevis
Source: Front Plant Sci. 2022 Feb 15;13:826158. doi: 10.3389/fpls.2022.826158 (PMC8886209; doi:10.3389/fpls.2022.826158)
Supplement: Supplementary file 2 [file Table_2.docx]

**Supplementary Table 2** Occurence of 32 haplotypes in analysed samples**.**

| **Haplotypes** | **Occurrence** | **Samples** |
| --- | --- | --- |
| Hap_1 | 3 | U1, U3, U42 |
| Hap_2 | 1 | U19 |
| Hap_3 | 1 | U37 |
| Hap_4 | 1 | U38 |
| Hap_5 | 1 | U21 |
| Hap_6 | 2 | U40, Us9 |
| Hap_7 | 2 | Us1, Us10 |
| Hap_8 | 2 | Us3, Us4 |
| Hap_9 | 2 | Us5, Us6 |
| Hap_10 | 6 | U10, U20, U30, U33, U41, U44 |
| Hap_11 | 1 | U26 |
| Hap_12 | 1 | U13 |
| Hap_13 | 1 | U8 |
| Hap_14 | 1 | U11 |
| Hap_15 | 1 | U2 |
| Hap_16 | 11 | U17, U27, U28, U29, U31, U34, U36, U4, U43, U45, U9 |
| Hap_17 | 1 | U25 |
| Hap_18 | 1 | U32 |
| Hap_19 | 1 | U39 |
| Hap_20 | 1 | Us2 |
| Hap_21 | 1 | U5 |
| Hap_22 | 1 | U6 |
| Hap_23 | 1 | U7 |
| Hap_24 | 1 | U12 |
| Hap_25 | 2 | Us7, Us8 |
| Hap_26 | 1 | U14 |
| Hap_27 | 1 | U15 |
| Hap_28 | 1 | U16 |
| Hap_29 | 1 | U18 |
| Hap_30 | 1 | U22 |
| Hap_31 | 1 | U23 |
| Hap_32 | 1 | U24 |
