# Supplementary material for: Novel Insights Into Refugia at the Southern Margin of the Distribution Range of the Endangered Species Ulmus laevis
Source: Front Plant Sci. 2022 Feb 15;13:826158. doi: 10.3389/fpls.2022.826158 (PMC8886209; doi:10.3389/fpls.2022.826158)
Supplement: Supplementary file 3 [file Table_3.docx]

**Supplementary Table S3** Estimates of haplotype (hapDiv) and nucleotide diversity (nucDiv) in cpDNAs of *U. laevis* populations, obtained separately by SNPs and InDels.

|  | SNPs | | InDels | |
| --- | --- | --- | --- | --- |
|  | nucDiv | hapDiv | nucDiv | hapDiv |
| France_SW | 0,00001 | 0,80000 | 0,52000 | 0,90000 |
| France_NE | 0,00000 | 0,46400 | 0,26548 | 0,96400 |
| Serbia | 0,00003 | 0,75800 | 0,39494 | 1,00000 |
| Italy | 0,00000 | 0,28100 | 0,29883 | 0,80100 |
| Spain | 0,00000 | NA | 0,47729 | 0,91100 |
| All | 0,00001 | 0,47100 | 0,15224 | 0,94300 |
